# Mental Health Utilization Among Transgender Veterans

**DOI:** 10.1001/jamanetworkopen.2024.54694

**Published:** 2025-01-13

**Authors:** Joy L. Lee, Adam Hirsh, Archana Radhakrishnan, Guneet K. Jasuja, Stanley Taylor, Stephanie Dickinson, Jocelyn Mineo, Jennifer Carnahan, Michael Weiner

**Affiliations:** 1 VA Center for Health Information and Communication, US Department of Veterans Affairs, Veterans Health Administration, Health Systems Research CIN 13-416, Richard L. Roudebush VA Medical Center, Indianapolis, Indiana; 2 VA Center for Healthcare Optimization and Implementation Research, US Department of Veterans Affairs, Veterans Health Administration, VA Bedford Healthcare System, Bedford, Massachusetts; 3 Department of Population and Quantitative Health Sciences, UMass Chan Medical School, Worcester, Massachusetts; 4 The Regenstrief Institute, Inc, Indianapolis, Indiana; 5 Department of Psychology, Indiana University Indianapolis, Indianapolis; 6 Department of Internal Medicine, University of Michigan, Ann Arbor; 7 VA Center for Clinical Management Research, VA Ann Arbor Healthcare System, Ann Arbor, Michigan; 8 Section of General Internal Medicine, Boston University Chobanian and Avedisian School of Medicine, Boston, Massachusetts; 9 Department of Health Law, Policy and Management, School of Public Health, Boston University, Boston, Massachusetts; 10 Department of Epidemiology and Biostatistics, School of Public Health, Indiana University Bloomington, Bloomington; 11 Department of Medicine, Indiana University School of Medicine, Indianapolis

## Abstract

**Importance:**

Compared with cisgender (CG) individuals, transgender and gender-diverse (TGD) individuals experience substantial social and economic disparities that can result in adverse mental health consequences. It is critical to understand potential barriers to care and to address the causes of the disparities in the future.

**Objective:**

To characterize mental health care utilization among TGD veterans with depression.

**Design, Setting, and Participants:**

This cohort study used electronic health record data from the US Department of Veterans Affairs (VA) to create a 1:3 age group–matched and VA facility–matched nationwide cohort of TGD and CG veterans with documentation of depression during 2018 to 2020. Data analysis was performed from January to November 2023.

**Exposure:**

TGD identity was ascertained by diagnosis of a gender identity disorder.

**Main Outcomes and Measures:**

The primary outcome was mental health care utilization, including counts of outpatient (in specialty care and primary care settings), telehealth, emergency department, and inpatient visits in this cohort. Descriptive statistics were used to characterize counts of mental health utilization, and statistically significant differences between TGD and CG veterans were tested using χ^2^ and Fisher exact tests. Wilcoxon rank-sum tests were used to test for differences in utilization between the 2 groups. Adjusted logistic regression, controlling for age group, administrative sex, race, Charlson Comorbidity Index, and number of mental health medications (eg, antidepressant, antipsychotic, and anxiolytic medications), was also used to compare utilization between TGD and CG veterans.

**Results:**

Among 10 564 veterans with depression (mean [SD] age, 46.4 [15.2] years; 8050 male [76.2%]), 2643 TGD veterans were matched with 7921 CG veterans. TGD veterans had 6 more specialty mental health visits per year than CG veterans (mean [SD], 13.93 [20.08] vs 8.46 [14.96] visits a year; median [range], 7.14 [0.00-246.30] vs 3.76 [0.00-202.38] visits per year). In adjusted models, compared with CG veterans, TGD veterans were 2.6 times more likely to have an outpatient mental-health visit (odds ratio, 2.60; 95% CI, 2.16-3.15).

**Conclusions and Relevance:**

In this cohort study of veterans with depression, TGD veterans had significantly higher utilization of mental health services compared with CG veterans, even after adjusting for several relevant health factors. Different health system resources may be required to meet the needs of this population. Further studies are needed to understand the determinants of these disparities and subsequently how to address them.

## Key Points

**Question:**

Is there a disparity between how transgender and gender-diverse (TGD) veterans with depression use mental health services compared with cisgender (CG) veterans?

**Findings:**

In this cohort study of 10 564 veterans, TGD veterans had significantly higher utilization of mental health services compared with CG veterans, even after adjusting for several relevant health factors. The difference was especially stark among specialty mental health visits: TGD veterans had 6 more visits per year compared with CG veterans.

**Meaning:**

These findings suggest that TGD and CG veterans have different patterns of mental health utilization and that different health system resources may be required to meet the needs of TGD veterans with depression.

## Teaser

This cohort study examines mental health care utilization among transgender and gender-diverse vs cisgender veterans with depression.

## Introduction

Transgender and gender-diverse (TGD) individuals experience substantial health care disparities because of socioeconomic disadvantage, discrimination, and stigma. These social and economic differences often translate to adverse mental health consequences, including higher rates of depression, anxiety, posttraumatic stress disorder, and substance use disorder compared with cisgender (CG) individuals.^1,2^ Not only are these disparities important in their own right, they also are associated with morbidity, decreased quality of life, and increased suicidality.^3^ In addition to experiencing known health disparities, many TGD patients also experience barriers to care. TGD patients report fewer primary care visits than do CG patients, with many citing fears of mistreatment as a reason.^4^ Understanding mental health care utilization by TGD veterans is important for determining whether they are receiving the care necessary to address their unique mental health concerns.

The US Department of Veterans Affairs (VA) is the largest integrated health care system in the US. It has stated its commitment to providing care for veterans “in a manner that is consistent with their self-identified gender identity”^5^ and standardizing health care services for TGD veterans. Yet many gaps in knowledge about the experiences of TGD veterans within the VA still exist, hampering efforts to improve care for this population.

Much of what is known about the mental health care of TGD veterans has come from surveys, including nationally representative surveys and veteran-specific surveys.^6,7,8^ Using data from the Behavioral Risk Factor Surveillance System to compare the health of TGD and CG veterans and civilians, Downing et al^2^ found that, although TGD and CG veterans had similar access to care, TGD veterans had higher odds of having at least 1 disability compared with CG veterans. In a national survey of TGD veterans, Lehavot et al^7^ found that 31% of respondents reported dissatisfaction with VA mental health care. Compared with TGD women, TGD men were more likely to report being dissatisfied (26% vs 56%).

Although survey findings can provide insights about TGD veterans’ perspectives on VA mental health care, an important, but less studied, complement to these surveys is the electronic health record (EHR). Data from the EHR can compare mental health care utilization patterns of TGD and CG veterans. In this project, we seek to compare the mental health care utilization of TGD vs CG veterans with depression. This is important because untreated depression can lead to long-term adverse consequences, including suicide.^9^ This investigation is a critical first step toward detecting disparities between TGD and CG veterans with depression, and working toward understanding and addressing the causes of the disparities in the future.

## Methods

We conducted a retrospective cohort analysis of VA medical records among veterans receiving care from calendar years 2018 to 2020. This study was approved by the Indiana University institutional review board and the Research & Development Committee of Roudebush VA Medical Center. An exemption for informed consent was granted given the secondary nature of the data analysis, in accordance with 45 CFR §46. This study adhered to the Strengthening the Reporting of Observational Studies in Epidemiology (STROBE) reporting guidelines for cohort studies.

### Data Source

The VA Central Data Warehouse inpatient and outpatient data files were used to characterize the cohort (including age, race, and ethnicity) and to ascertain cohort eligibility, including TGD identification, depression diagnosis, and receipt of care at the VA. The Central Data Warehouse includes data on demographics, diagnoses, health care encounters, orders including prescriptions, consultations, diagnostic test results, and procedures.

### Depression Diagnosis

We used *International Statistical Classification of Diseases and Related Health Problems, Tenth Revision (ICD-10)* codes to identify eligible veterans with depression. Veterans had to have a depression diagnosis during the study period, no documented diagnosis of depression in the prior 12 months, and at least 1 documented outpatient primary care or mental health visit 12 months prior to depression diagnosis (see eTable 1 in Supplement 1 for relevant diagnosis codes). Table 1 explains the eligibility criteria in detail.

**Table 1.** Eligibility Criteria for Study Sample

Eligibility criteria	Time frame	Definition
Transgender and gender-diverse identity	Any time	At least 1 documented diagnosis of gender identity disorder^5^
Depression	January 1, 2017-December 31, 2018	At least 1 documented diagnosis of depression during eligibility time frame, and no documented diagnosis of depression in the prior 12 mo (to minimize unmeasured variation in the duration of diagnosis)
Receipt of care at the Veterans Health Administration	1 y (12 mo) Before depression diagnosis	At least 1 documented outpatient primary care or mental health visit

### TGD Sample

We used *ICD-10* codes for gender identity disorders to identify the majority of TGD veterans. Although a self-identified gender identity field was available, the incompleteness of the data at the time of our data collection led us to use *ICD-10* codes. A small number of veterans were identified by VA Health Factors data (4.7%) with relevant TGD labels.^10^ *ICD-10* diagnosis codes are prerequisites for accessing some gender-affirming treatments for TGD veterans, including gender-affirming hormone therapy, but not mental health services.^5^ Although self-report remains the reference standard for identifying TGD veterans, a validation study by Blosnich et al^11^ found that TGD-related codes had high sensitivity.

### Matching CG Sample

We used a 3-to-1 matching strategy to identify the CG control sample. The sample has the same eligibility criteria as the TGD veterans except for the diagnosis of gender identity disorder. Veterans were matched on age group by decade and the facility where they received care during the eligibility time frame. For patients who may have been in 2 age groups within the time frame or sought care in 2 facilities, we used the first age group or first facility for matching.

### Measures

We controlled for patient characteristics available in the medical record, including sex, age group, race (American Indian or Alaska Native, Asian, Black or African American, Native Hawaiian or Other Pacific Islander, White, or unknown), Hispanic ethnicity, VA medical center, and VA region (Continental, Midwest, North Atlantic, Pacific, and Southeast). Data on race and ethnicity are included in this analysis to assess whether the distribution of these variables was similar between the TGD and CG cohorts. For each patient, we calculated the Charlson Comorbidity Index (CCI)^12^ and the number of mental health medications prescribed to the patient (antidepressants, antipsychotics, and anxiolytics; see eTable 2 in Supplement 1 for full list). Medication counts were grouped as low (0-1 prescribed), medium (>1 to 10 prescribed), and high (>10 prescribed). Our outcomes of interest were mental health care utilization, by setting, during the 24 months following a patient’s depression diagnosis within the eligibility time frame (2018-2020). The outcome window was rolling and may differ for each eligible patient. Types of mental health care utilization were validated by EHR review to assess whether diagnosis codes, note titles, and stop codes corresponded with the primary reason for the patients’ encounters. We reviewed equal numbers of a random set of TGD and CG patient EHRs (approximately 2% of the total sample) to examine elements related to mental health ascertainment for different types of utilization. We defined utilization per year in the 4 ways outlined in the following subsections.

#### Mental Health Encounters 

Mental health encounters included in-person outpatient encounters with a mental health care practitioner (psychologist, psychiatrist, mental health counselor, or behavioral health social worker) not in primary care. These encounters were identified by EHR note titles and stop codes.

#### Primary Care Encounter (Mental Health and Non–Mental Health) 

Primary care encounters were defined as in-person, outpatient primary care encounters. Primary care designation was ascertained by stop codes. Encounters were categorized as mental health related or not according to note titles and primary diagnosis codes.

#### Telehealth Encounters 

Telehealth encounters were defined as documented telephone, secure messaging, or video contact notes in the EHR. These encounters were noted as mental health ones if conducted with a mental health practitioner according to stop codes.

#### Hospital Encounters 

Hospital encounters included inpatient stays and emergency department (ED) visits. These were categorized as mental health related or not according to the primary discharge diagnosis and primary diagnosis, respectively.

### Statistical Analysis

Data analysis was performed from January to November 2023. We used descriptive statistics (eg, mean and median) to report the estimated health care utilization rates for TGD veterans with depression. We used descriptive statistics to characterize counts of mental health utilization and tested for statistically significant differences between TGD and CG veterans using χ^2^ and Fisher exact tests. Wilcoxon rank-sum tests were used to test for differences in utilization between the 2 groups. Statistical significance was set at 2-sided *P* < .05. In separate logistic regression models, we considered the veteran characteristics related to 4 types of mental health utilization, allowing for comparisons between TGD veterans and matched CG veterans on utilization of health care (yes vs no), controlling for patient age group, sex, race, CCI, number of prescribed mental health–related medications, and VA district. Random effects were used to account for clustering within facilities. We used SAS Enterprise statistical software version 8.3 (SAS Institute) for data management and R statistical software version 4.4.1 (R Project for Statistical Computing) to analyze the data.

## Results

The cohort of 10 564 veterans (mean [SD] age, 46.4 [15.2] years; 8050 male [76.2%]) included 2643 TGD veterans and 7921 matched CG veterans. Table 2 shows the characteristics of this matched sample. With regard to race and ethnicity, 122 veterans (1.2%) were American Indian or Alaska Native, 162 (1.5%) were Asian, 1921 (18.2%) were Black or African American, 876 (8.3%) were Hispanic, 106 (1.0%) were Native Hawaiian or Other Pacific Islander, 7543 (71.4%) were White, and 710 (6.7%) were of unknown race. Significant differences were noted between TGD and CG groups in terms of age, race, gender, CCI, and mental health–related medications. Although TGD and CG veterans were matched by age group, the TGD group was younger than the CG group (mean [SD] age, 45.3 [15.5] years vs 46.8 [15.1] years). Consistent with prior VA studies, the TGD group had significantly more White veterans (2059 veterans [77.9%] vs 5484 veterans [69.2%]).^1,13,14^ The TGD group also had significantly more female veterans (1072 veterans [40.6%] vs 1442 veterans [18.2%]). On average, the TGD group had lower CCI than the CG group, but the difference was small (mean [SD], 0.86 [1.57] vs 0.89 [1.76]; median [range], 0.0 [0.0-15.0] vs 0.0 [0.0-14.0]). Most veterans (2420 TGD veterans [90.3%] and 7154 CG veterans [91.6%]) had at least 1 primary care visit per year during the study period.

**Table 2.** Matched Patient Characteristics

Characteristics	Patients, No. (%)			*P* value
	Overall (N = 10 564)	Cisgender (n = 7921)	Transgender and gender diverse (n = 2643)	
Age, y				
Mean (SD)	46.4 (15.2)	46.8 (15.1)	45.3 (15.5)	<.001
Median (range)	45.0 (18.0-98.0)	46 (18.0-98.0)	44 (18.0-97.0)	
Age group, y				
<21	12 (0.1)	7 (0.1)	5 (0.2)	.93
21-30	2528 (23.9)	1896 (23.9)	632 (23.9)	
31-40	2284 (21.6)	1713 (21.6)	571 (21.6)	
41-50	1520 (14.4)	1140 (14.4)	380 (14.4)	
51-60	1884 (17.8)	1413 (17.8)	471 (17.8)	
61-70	1920 (18.1)	1440 (18.2)	480 (18.2)	
>70	416 (3.9)	312 (3.9)	104 (3.9)	
Administrative sex				
Female	2514 (23.8)	1442 (18.2)	1072 (40.6)	<.001
Male	8050 (76.2)	6479 (81.8)	1571 (59.4)	
Race				
American Indian or Alaska Native	122 (1.2)	89 (1.1)	33 (1.2)	<.001
Asian	162 (1.5)	119 (1.5)	43 (1.6)	
Black or African American	1921 (18.2)	1599 (20.2)	322 (12.2)	
Native Hawaiian or Other Pacific Islander	106 (1.0)	86 (1.1)	20 (0.8)	
White	7543 (71.4)	5484 (69.2)	2059 (77.9)	
Unknown	710 (6.7)	544 (6.9)	166 (6.3)	
Hispanic				
No	9304 (88.1)	6933 (87.5)	2371 (89.7)	<.001
Yes	876 (8.3)	692 (8.7)	184 (7.0)	
Unknown	384 (3.6)	296 (3.7)	88 (3.3)	
District				
Continental	1960 (18.6)	1470 (18.6)	490 (18.5)	>.99
Midwest	1991 (18.8)	1493 (18.8)	498 (18.8)	
North Atlantic	2012 (19.0)	1509 (19.1)	503 (19.0)	
Pacific	3086 (29.2)	2313 (29.2)	773 (29.2)	
Southeast	1515 (14.3)	1136 (14.3)	379 (14.3)	
Charlson Comorbidity Index				
Mean (SD)	Not applicable	0.89 (1.46)	0.86 (1.57)	<.001
Median (range)	Not applicable	0.0 (0.0-14.0)	0.0 (0.0-15.0)	

Table 3 presents the mental health utilization patterns of TGD and CG veterans. These include visits to mental health practitioners and primary care practitioners; telephone, video, and secure message contacts between veterans and mental health practitioners; and hospital utilization in the form of inpatient stays and ED visits.

**Table 3.** Mental Health Utilization

Encounter type	No. of visits per year				*P* value
	Cisgender		Transgender and gender diverse		
	Median (range)	Mean (SD)	Median (range)	Mean (SD)	
Outpatient					
Mental health in-person	3.76 (0.00-202.38)	8.46 (14.96)	7.14 (0.00-246.3)	13.93 (20.08)	<.001
Mental health telephone call	0.34 (0.00-59.42)	1.03 (2.39)	0.57 (0.00-60.62)	1.59 (3.29)	<.001
Mental health video	0.00 (0.00-25.40)	0.29 (1.25)	0.00 (0.00-25.08)	0.38 (1.92)	.09
Mental health secure message	0.00 (0.00-12.94)	0.02 (0.28)	0.00 (0.00-4.50)	0.02 (0.2)	.07
Primary care mental health	0.00 (0.00-17.65)	0.05 (0.43)	0.00 (0.00-16.32)	0.06 (0.46)	.19
Primary care non–mental health	1.96 (0.00-97.33)	2.63 (3.36)	2.12 (0.00-34.25)	2.76 (2.68)	<.001
Telehealth					
Secure messaging	0.00 (0.00-73.71)	0.45 (2.88)	0.00 (0.00-60.00)	0.54 (2.67)	.14
Telephone calls	1.76 (0.00-172.64)	3.62 (6.55)	2.4 (0.00-61.25)	4.25 (5.82)	<.001
Video visits	0.00 (0.00-26.55)	0.49 (1.51)	0.00 (0.00-28.95)	0.64 (2.23)	<.001
Hospital and ED					
ED visit (mental health)	0.00 (0.00-21.94)	0.10 (0.58)	0.00 (0.00-12.92)	0.13 (0.57)	.42
ED visit (non–mental health)	0.00 (0.00-76.27)	0.88 (2.63)	0.00 (0.00-44.45)	0.96 (2.1)	<.001
Inpatient stay (mental health)	0.00 (0.00-18.95)	0.12 (0.59)	0.00 (0.00-10.86)	0.17 (0.62)	.02
Inpatient stay (non–mental health)	0.00 (0.00-33.96)	0.21 (1.15)	0.00 (0.00-16.49)	0.15 (0.69)	.67

Abbreviation: ED, emergency department.

### Outpatient Mental Health Utilization

In-person visits with mental health practitioners were common among both TGD and CG veterans: 2489 veterans (94.2%) of the TGD group had at least 1 in-person encounter with a mental health practitioner in a year, over the 2 years after depression diagnosis, as had 6978 veterans (88.1%) of the CG group. On average, veterans in the TGD group had significantly more in-person encounters than the CG group (mean [SD], 13.93 [20.08] vs 8.46 [14.96] visits a year; median [range], 7.14 [0.00-246.30] vs 3.76 [0.00-202.38] visits per year). The TGD group had more primary care encounters than the CG group, although only the difference between non–mental health primary care visits was statistically significant.

### Telehealth Utilization

During the study period between 2018 and 2020, telephone encounters were the most common type of telehealth for both the TGD and CG groups. More than 80% of veterans in our sample made at least 1 documented telephone call a year. The TGD group had significantly more calls, on average, than the CG group (mean [SD], 4.25 [5.82] vs 3.62 [6.55] calls) and significantly more video visits as well (mean [SD], 0.64 [2.23] vs 0.49 [1.51] visits).

### Hospital and ED Utilization

Non–mental health–related visits were more common in both ED and inpatient stays than mental health–related visits and stays. Despite being infrequent (<20% of either group had these types of visits), the TGD group had on average, more ED visits and inpatient stays than the CG group. The differences between the 2 groups were statistically significant for all categories except non–mental health ED visits.

### Factors Associated With In-Person Mental Health Visits

Table 4 shows the association between veteran characteristics and their use of mental health services in the VA. In multivariable adjusted models, we observed that TGD veterans were more likely than CG veterans to have mental health visits across all utilization categories. Race and ethnicity were not consistently associated with mental health visits in this cohort; Hispanic veterans were more likely to have outpatient mental health visits and primary care non–mental health visits than non-Hispanic veterans, but not for other types of mental health utilization. The odds of a TGD veteran having an outpatient mental health visit were more than twice those of a CG veteran (odds ratio [OR], 2.60; 95% CI, 2.16-3.15). Although significant, the ORs for TGD veterans vs CG veterans were slightly lower for the other types of visits, ranging from 1.24 (95% CI, 1.05-1.46) for non–mental health primary care visits to 1.65 (95% CI, 1.44-1.89) for inpatient mental health care. The odds of veterans having mental health visits decreased with age, so that veterans aged 31 years or older were less likely to have outpatient mental health visits than those younger than 31 years. A similar trend could be observed for ED and inpatient mental health visits, although the trend did not hold for primary care visits. Veterans with a CCI of 1 had an OR of 1.28 (95% CI, 1.07-1.53) for a mental health visit, compared with those with a CCI of 0. Veterans prescribed fewer medications also had lower odds of having mental health visits.

**Table 4.** Patient Factors Associated With Mental Health Care Utilization

Characteristics	OR (95% CI)				
	Outpatient mental health	Primary care (mental health)	Primary care (non–mental health)	ED mental health	Inpatient mental health
Age group, y					
<31	1 [Reference]	1 [Reference]	1 [Reference]	1 [Reference]	1 [Reference]
31-40	0.74 (0.60-0.92)	1.14 (0.86-1.50)	1.44 (1.20-1.73)	0.81 (0.67-0.96)	0.79 (0.66-0.94)
41-50	0.58 (0.46-0.73)	1.09 (0.80-1.49)	1.17 (0.95-1.45)	0.68 (0.55-0.84)	0.63 (0.52-0.77)
51-60	0.55 (0.44-0.70)	1.20 (0.90-1.62)	1.29 (1.04-1.60)	0.73 (0.60-0.89)	0.88 (0.73-1.06)
61-70	0.40 (0.32-0.50)	1.19 (0.88-1.62)	1.35 (1.06-1.73)	0.37 (0.29-0.47)	0.37 (0.30-0.47)
>70	0.22 (0.16-0.29)	1.25 (0.77-1.98)	1.47 (0.96-2.35)	0.31 (0.19-0.49)	0.21 (0.12-0.35)
Administrative sex					
Female	1 [Reference]	1 [Reference]	1 [Reference]	1 [Reference]	1 [Reference]
Male	1.17 (0.99-1.38)	0.87 (0.71-1.06)	1.06 (0.90-1.24)	1.30 (1.11-1.52)	1.55 (1.33-1.80)
Gender identity					
Cisgender	1 [Reference]	1 [Reference]	1 [Reference]	1 [Reference]	1 [Reference]
Transgender and gender diverse	2.60 (2.16-3.15)	1.38 (1.14-1.68)	1.24 (1.05-1.46)	1.54 (1.34-1.78)	1.65 (1.44-1.89)
Race					
American Indian or Alaska Native	1 [Reference]	1 [Reference]	1 [Reference]	1 [Reference]	1 [Reference]
Asian	2.00 (0.78-5.41)	2.04 (0.57-9.51)	0.68 (0.30-1.45)	1.57 (0.68-3.87)	0.64 (0.27-1.51)
Black or African American	1.47 (0.73-2.72)	2.70 (0.99-11.1)	1.02 (0.50-1.89)	1.83 (0.95-3.99)	1.45 (0.81-2.80)
Native Hawaiian or Other Pacific Islander	0.66 (0.28-1.55)	0.82 (0.11-5.04)	1.06 (0.43-2.64)	0.78 (0.25-2.28)	0.95 (0.38-2.32)
Unknown	0.83 (0.41-1.58)	1.96 (0.67-8.37)	0.88 (0.42-1.70)	1.30 (0.64-2.92)	1.12 (0.60-2.24)
White	0.83 (0.42-1.49)	2.43 (0.91-9.92)	1.04 (0.52-1.88)	1.36 (0.72-2.94)	1.15 (0.65-2.20)
Ethnicity					
Non-Hispanic	1 [Reference]	1 [Reference]	1 [Reference]	1 [Reference]	1 [Reference]
Hispanic	1.36 (1.05-1.78)	0.85 (0.59-1.20)	1.30 (1.01-1.71)	1.23 (0.97-1.54)	0.88 (0.69-1.11)
Region					
Continental	1 [Reference]	1 [Reference]	1 [Reference]	1 [Reference]	1 [Reference]
Midwest	1.07 (0.87-1.33)	1.51 (1.10-2.09)	0.84 (0.66-1.07)	1.22 (0.98-1.53)	1.51 (1.24-1.84)
North Atlantic	1.19 (0.96-1.48)	1.43 (1.04-1.98)	0.66 (0.53-0.83)	1.71 (1.39-2.11)	1.38 (1.14-1.69)
Pacific	1.05 (0.86-1.27)	2.20 (1.66-2.96)	0.68 (0.55-0.84)	1.15 (0.94-1.42)	1.12 (0.93-1.36)
Southeast	1.43 (1.11-1.83)	1.64 (1.17-2.29)	0.84 (0.65-1.08)	1.42 (1.13-1.78)	0.93 (0.74-1.17)
Charlson Comorbidity Index					
0	1 [Reference]	1 [Reference]	1 [Reference]	1 [Reference]	1 [Reference]
1	1.28 (1.07-1.53)	1.30 (1.04-1.63)	2.66 (2.16-3.31)	1.13 (0.96-1.34)	1.20 (1.03-1.39)
≥2	1.08 (0.90-1.31)	1.55 (1.22-1.97)	2.68 (2.11-3.43)	1.15 (0.94-1.40)	0.98 (0.81-1.18)
Medication count					
Medium	1 [Reference]	1 [Reference]	1 [Reference]	1 [Reference]	1 [Reference]
Low	0.20 (0.17-0.23)	0.68 (0.56-0.82)	0.42 (0.37-0.49)	0.44 (0.38-0.50)	0.38 (0.33-0.44)
High	2.03 (0.92-5.75)	1.84 (1.16-2.79)	0.64 (0.40-1.08)	3.26 (2.42-4.36)	4.93 (3.73-6.51)

Abbreviations: ED, emergency department; OR, odds ratio.

## Discussion

Health care utilization was high in this cohort study of more than 10 000 matched veterans with depression: most TGD and CG veterans had at least 1 primary care visit (90.3% and 91.6%, respectively) and outpatient mental health visit (94.2% and 88.1%, respectively) a year during the study period. We observed that TGD veterans had higher use of mental health services compared with CG veterans. The starkest difference was in outpatient mental health visits, where TGD veterans had 6 more encounters per year, on average, compared with CG veterans. Even after adjusting for demographic variables (eg, age, sex, and race), and measures of health (eg, number of mental health medications and CCI). We also found that TGD veterans also had more documented telephone calls and video visits in this pre–COVID-19 cohort.

One possible interpretation of our findings is that TGD veterans have greater mental health need than do CG patients. This interpretation is supported by findings from Livingston et al,^1^ who found, using VA EHR data, that the prevalence of depression, posttraumatic stress disorder, schizophrenia, substance use disorder, and military sexual trauma were all higher among TGD veterans than CG veterans. The greater use of services was especially evident in mental health specialty visits, where we observed the greatest differences between TGD and CG veterans. Although we did observe statistically significant differences in primary care mental health visits, ED mental health visits, and inpatient mental health stays between TGD and CG veterans, the magnitude of these differences was smaller and of questionable clinical importance. The differences between TGD and CG veterans may also point to lower treatment effectiveness, greater recurrence of symptoms of TGD veterans, or different preferences for care between TGD and CG veterans. Mental health services, especially in the outpatient setting, might also be less stigmatized in the TGD population, leading to more willingness to access care, compared with CG veterans. Although our findings do not directly indicate whether the needs of either TGD or CG veterans with depression are unmet, they indicate the need for more research to explore the differences.

Telehealth tools, such as patient portals, telephone visits, and video visits, have all been suggested to overcome access barriers for underserved patient populations,^15,16,17,18^ although little prior research has focused specifically on telehealth use among the adult TGD population.^19^ The use of telehealth tools within the VA drastically increased during the COVID-19 pandemic; its application in mental health has been especially robust.^20,21,22^ Our finding of higher use of telephone calls and video visits among TGD veterans suggests perhaps greater willingness to engage with telehealth tools or need for access to mental health services. Future research could explore the potential of telehealth tools in addressing access barriers and enhancing mental health care delivery to TGD and CG veterans.

The higher rates of utilization among TGD veterans with depression may seem counterintuitive given known health care access issues and health care avoidance for TGD patients from surveys. For example, in the civilian population, Rusow et al^23^ found that TGD youths have less access to health care and higher depression burden compared with CG youths. However, our findings are in line with other studies of patients with depression using EHR data. Reisner et al^24^ observed that TGD youths have higher rates of depression and other mental health outcomes and higher rates of both inpatient and outpatient mental health treatment use vs CG youths. In a study of Swedish individuals using data from a national register, Bränström et al^25^ found that TGD individuals were approximately 6 times as likely to have had a health care visit because of a mood or anxiety disorder and more than 3 times as likely to have received prescriptions for antidepressants and anxiolytic medications compared with CG individuals. Although those studies focused on the civilian population, the findings indicate a high health burden experienced by TGD patients with depression.

### Limitations

In this study, we were limited by the available data characterizing TGD and CG patients during our study period. Although the TGD and CG veterans were matched by geography (to account for site differences in treatment) and age group, the TGD sample differed from the CG sample on a few key demographic factors. The difference in administrative sex was especially noteworthy. At the time of our data collection, gender identity was not routinely or widely collected in the VA.^10^ Hence, veterans could update the administrative sex field—initially reflecting sex assigned at birth—to reflect their inferred gender identity. This administrative sex variable in the medical record was used to represent interrelated constructs of inferred gender identity and sex assigned at birth. It is, thus, unclear whether the observed gender differences in our study represent differences in gender identity or sex assigned at birth between the 2 groups. Because self-identified gender identity data were not widely and routinely collected in the VA during the start of our start study period, we are also unable to disaggregate TGD patients who identify as transfeminine, transmasculine, or nonbinary. Limited research suggests heterogeneity of mental health experiences and utilization patterns among this population. In 1 VA survey, transmasculine veterans were more than 3 times as likely to report dissatisfaction with VA care than transfeminine veterans.^7^ In civilian TGD adults, Reisner et al^24^ observed lower utilization among nonbinary TGD civilian adults compared with TGD adults with binary identity.

## Conclusions

In this cohort study of veterans with depression, we found that TGD veterans had 6 more mental health specialist visits per year compared with a matched cohort of CG veterans. Even after matching for patient characteristics, the odds of having a mental health specialist visit for TGD veterans were 2.6 times that for CG veterans. Our findings are among the first to describe the mental health utilization patterns of TGD veterans and to demonstrate a disparity between TGD and CG veterans with depression using EHR data. Prior research had highlighted differences in mental health outcomes between TGD and CG veterans, as well as reports of mistreatment from TGD veterans.^2,6,7,8^ Building on this work, we describe how TGD veterans use mental health services in the VA. The results of this study are noteworthy for explicitly highlighting the role of gender identity in the mental health care utilization of a matched cohort of veterans with depression. The differences between the 2 groups may indicate that TGD veterans have greater depression severity than CG veterans. More research is needed to understand the cause of these differences and, especially, how to best address the mental health concerns and experiences of TGD veterans.

## Notes

Supplement 1. **eTable 1.** Relevant *ICD* codes for depression and gender identity disorder **eTable 2.** Relevant psychotropic prescription drugs

Supplement 2.
Data Sharing Statement
