# Supplementary material for: Mental Health Utilization Among Transgender Veterans
Source: JAMA Netw Open. 2025 Jan 13;8(1):e2454694. doi: 10.1001/jamanetworkopen.2024.54694 (PMC11731220; doi:10.1001/jamanetworkopen.2024.54694)
Supplement: Supplement 1. — eTable 1. Relevant ICD codes for depression and gender identity disorder eTable 2. Relevant psychotropic prescription drugs [file jamanetwopen-e2454694-s001.pdf]

Supplemental Online Content

Lee JL, Hirsh A, Radhakrishnan A, et al. Mental health utilization among transgender veterans. *JAMA Netw Open*. 2025;8(1):e2454694.
doi:10.1001/jamanetworkopen.2024.54694

eTable 1. Relevant *ICD* codes for depression and gender identity disorder

eTable 2. Relevant psychotropic prescription drugs

This supplemental material has been provided by the authors to give readers additional information about their work.

© 2025 Lee JL et al. *JAMA Network Open*.

eTable 1: Relevant *ICD* codes for depression and gender identity disorder

Depression:

F31.30; F31.31; F31.32; F31.4; F31.5; F31.60; F31.61; F31.62; F31.63; F31.64; F31.75; F31.76; F31.77; F31.78; F31.81; F32.0; F32.1; F32.2; F32.3; F32.4; F32.5; F32.9; F33.0; F33.1; F33.2; F33.3; F33.40; F33.41; F33.42; F33.8; F33.9; F34.1; F43.21; F43.23

Gender identity disorder:

F64.0; F64.1; F64.2; F64.8; F64.9

© 2025 Lee JL et al. *JAMA Network Open*.

eTable 2: Relevant psychotropic prescription drugs

<u>Antidepressants</u>

Selective serotonin reuptake inhibitors (SSRI)

citalopram

escitalopram

fluoxetine

fluvoxamine

paroxetine

sertraline

vilazodone (serotonin modulator)

vortioxetine (serotonin modulator)

Serotonin and norepinephrine reuptake inhibitors (SNRI)

desvenlafaxine

duloxetine

levomilnacipran

milnacipran

venlafaxine

Tricyclic antidepressants (TCA)

amitriptyline

amoxapine

clomipramine

desipramine

doxepin

imipramine

maprotiline

nortriptyline

protiptyline

trimipramine

Monoamine oxidase inhibitors (MAOI)

isocarboxazid

© 2025 Lee JL et al. *JAMA Network Open*.

phenelzine

selegiline

tranylcypromine

N-methyl-D-aspartate (NMDA) receptor antagonist

esketamine

Atypical antidepressants

bupropion (dopamine reuptake inhibitor)

mirtazapine (noradrenergic antagonist)

trazodone

vortioxetine

Other Psychotropic Medications

Typical antipsychotics

benperidol

chlorpromazine

fluphenazine

haloperidol

loxapine

mesoridazine

molindone

perphenazine

pimozide

thioridazine

thiothixene

trifluoperazine

Atypical antipsychotics

aripiprazole

asenapine

brexpiprazole

cariprazine

clozapine

© 2025 Lee JL et al. *JAMA Network Open*.

lurasidone

olanzapine

paliperidone

quetiapine

risperidone

Anxiolytics

alprazolam

atenolol

buspirone

chlordiazepoxide

clobazam

clonazepam

clorazepate

diazepam

estazolam

hydroxyzine

lorazepam

oxazepam

propranolol

temazepam

tiagabine

Hypnotics

chlordiazepoxide

eszopiclone

phenobarbital

suvorexant

zaleplon

zolpidem

zopiclone

Mood stabilizers

© 2025 Lee JL et al. *JAMA Network Open*.

carbamazepine
gabapentin
lamotrigine
levetiracetam
lithium
oxcarbazepine
valproic acid (sodium valproate)

Stimulants and non-stimulants for ADHD and related disorders

amphetamine and dextroamphetamine
atomoxetine
dextroamphetamine (sulfate)
lisdexamfetamine
methamphetamine (hydrochloride)
methylphenidate

For Alzheimer's disease or other dementias

donepezil
galantamine

Anti-seizure

topiramate

Recreational abuse

dextromethorphan

For alcohol or other substance dependence or withdrawal

disulfiram
chlordiazepoxide
naltrexone

© 2025 Lee JL et al. *JAMA Network Open*.
