# Supplementary material for: Mental Health Utilization Among Transgender Veterans
Source: JAMA Netw Open. 2025 Jan 13;8(1):e2454694. doi: 10.1001/jamanetworkopen.2024.54694 (PMC11731220; doi:10.1001/jamanetworkopen.2024.54694)
Supplement: Supplement 2. — Data Sharing Statement [file jamanetwopen-e2454694-s002.pdf]

Data Sharing Statement

Lee. Mental Health Utilization Among Transgender Veterans. *JAMA Netw Open*. Published January 13, 2025. doi:10.1001/jamanetworkopen.2024.54694

Data
Data available: No

Additional Information
Explanation for why data not available: Data sharing requests may be made by contacting the corresponding author.
